# Preoperative Anesthesia Assessment in Elderly Patients: Challenges and Opportunities for Safer Perioperative Care

**DOI:** 10.7759/cureus.89174

**Published:** 2025-07-31

**Authors:** María Luisa Alvarado Mora, María Laura Alvarado Fernández, Fiorella Apuy Rodríguez, María Jesús Arias Alvarado, Jessica Arias Valverde

**Affiliations:** 1 Faculty of Medicine, University of Costa Rica, San José, CRI

**Keywords:** cognitive impairment, delirium, elderly patients, frailty, postoperative complications, preoperative assessment

## Abstract

As the global population ages, older adults represent an increasing proportion of surgical patients. This demographic presents unique characteristics that increase their risk for postoperative complications, including delirium, functional decline, and mortality. Frailty has emerged as a key predictor of adverse outcomes, reflecting diminished functional and physiological reserves. Cognitive impairment, particularly when combined with frailty (cognitive frailty), further increases the risk, yet routine preoperative screening remains uncommon. This review examines the limitations of traditional preoperative evaluations in older adults and emphasizes the value of incorporating frailty and cognitive assessments using validated tools for more accurate risk stratification. Early identification of these factors may facilitate targeted interventions, ultimately improving postoperative recovery and functional recovery in this vulnerable population.

## Introduction and background

According to demographic projections, the global population over the age of 65 is expected to double within the next 30 years, while those over 85 will triple. This expanding demographic already accounts for more than 30% of all surgical procedures. In older adults, preoperative assessment must consider factors beyond standard risk evaluation, as this population is at increased risk of losing independence and experiencing postoperative functional decline. For example, individuals with known neurocognitive disorders have a significantly higher probability of developing postoperative delirium, as outlined in the American Society of Anesthesiologists (ASA) Practice Advisory for Perioperative Care of Older Adults. However, traditional preoperative assessments often fail to address age-related vulnerabilities such as frailty and cognitive impairment, limiting their ability to accurately predict postoperative risks in older adults [1].

Frailty is a central concept in this context; it reflects declines in both physical and physiological reserves due to chronic conditions and age-related deficits. These individuals experience multisystem decline, making them more susceptible to surgical stressors and increasing their risk of delirium, major morbidity, cognitive deterioration, and mortality [1-2].

Frailty assessment tools aid in preoperative risk stratification by evaluating physical function and overall health status. These tools can predict one-, three-, and 12-month mortality, postoperative complications, and length of hospital stay [3]. The Clinical Frailty Scale evaluates overall health, mobility, comorbidities, and cognitive function. The Fried Phenotype Scale, one of the most accepted tools, includes five criteria: weakness, slowness, low physical activity, exhaustion, and unintentional weight loss. The Edmonton Frailty Scale includes nine categories: cognition, health status, functional independence, social support, medication, nutrition, mood, continence, and functional performance [3-4].

## Review

Limitations of traditional preoperative assessment in the older population

Over time, standard methods have been used to stratify a patient’s risk before surgery; however, according to the ASA guidelines, various approaches may provide better postoperative outcomes. These include enhanced preoperative assessment, appropriate selection of anesthetic techniques, and optimal pharmacologic management. Enhanced assessment in the elderly population should focus on frailty, mental health issues, malnutrition, baseline functionality, and cognition, as well as polypharmacy [1].

Given the current limitations, some questions with different levels of evidence have been posed to optimize preoperative evaluation. For example, in patients with frailty or cognitive impairment, evidence suggests that an expanded approach like the Comprehensive Geriatric Assessment (CGA) combined with a multidisciplinary team may reduce the risk of delirium; however, the strength of the evidence was low due to reliance on a small study. Regarding anesthesia method selection, no significant differences were found in recovery outcomes between neuraxial and general anesthesia, nor between general anesthesia and inhaled anesthesia. Another question addressed the need for pharmacologic delirium prevention. On this topic, evidence indicated that it would be appropriate to consider using dexmedetomidine to lower the risk of delirium; however, caution is necessary due to its potential to increase bradycardia and hypotension [1].

Considerations regarding medication have also been researched; for instance, long-acting benzodiazepines have been shown to increase the incidence of delirium, whereas short-acting benzodiazepines appear to lower it. For other drugs, such as antipsychotics and ketamine, the evidence remains inconclusive. Another key aspect is prehabilitation, which is proposed to reduce the incidence of postoperative delirium through interventions like exercise, nutritional supplementation, and cognitive training [1].

Evidence regarding prehabilitation remains conflicting; for example, a 2025 systematic review and network meta-analysis by McIsaac et al. included 178 randomized controlled trials with a total of over 18,000 patients [5]. The study found that prehabilitation (including exercise, nutrition, and psych support) was associated with a statistically significant reduction in postoperative complications and length of hospitalization. However, the authors emphasized that further trials with lower risk of bias are needed to confirm these findings [5].

Another positive result is a recent meta-analysis by D’Amico et al., which included 29 randomized controlled trials and a total of 3,508 patients [6]. Home-based prehabilitation was associated with a significantly lower risk of postoperative complications (508/1,322 (38.4%) vs. 578/1,335 (43.3%); risk ratio (RR) 0.84, 95% CI: 0.72-0.98; P = 0.02), as well as improved preoperative functional capacity (CI 9.5-46.9; P < 0.01). Additionally, levels of anxiety and depression were significantly reduced (P < 0.001), and hospital length of stay was modestly shortened (CI -0.61 to -0.03; P = 0.03), although the certainty of evidence was low [6].

Cochrane reviews support prehabilitation in colorectal cancer surgery to increase functional capacity, reduce complications, and improve quality of life [7]. Another study based on major upper abdominal surgery is a 2024 systematic review and meta-analysis by Amirkhosravi et al., which included 10 randomized controlled trials encompassing 1,503 patients undergoing elective upper abdominal procedures [8]. The meta-analysis found significantly lower odds of all-cause postoperative complications (95% CI: -0.75 to -0.004; P =  0.048) compared to control groups. Among five studies reporting on postoperative pulmonary complications (PPCs), prehabilitation was associated with reduced risk (P < 0.001). Hospital length of stay was not significantly reduced, unless exercise was included; in trials including exercise, hospital stay decreased significantly (P = 0.02) [8]. On the other hand, seven randomized control trials showed that cognitive prehabilitation had no clear effect on either postoperative delirium or cognitive dysfunction, so evidence still lacks consistency [9]. The PREPARE trial, once published, may provide further clarity regarding cognitive prehabilitation efficacy [1].

The European Society of Anaesthesiology and Intensive Care (ESAIC) has also proposed certain guidelines for this population, though these are not yet widely implemented. According to the EISAC’s recommendations, the strongest evidence supports the inclusion of telemedicine and standardized questionnaires during preoperative assessments. Additionally, early outpatient evaluations (ideally within 30 days before surgery) are suggested to allow time for fitness improvement. Other recommendations involve consultations with relevant specialists to optimize underlying conditions, as well as specific assessments for airway management, renal function, and known coagulation disorders [3].

In high-risk patients, frailty testing is recommended to predict the likelihood of postoperative delirium. If a frailty phenotype is identified, the Clinical Frailty Scale should be applied, and a geriatric consultation is advised to address nutrition, comorbidities, and cognition. For patients at high risk of complications, decisions regarding prehabilitation and nutritional support should be made on a case-by-case basis [3].

Frailty as a predictor of postoperative risk

Frailty assessments are increasingly used as part of preoperative evaluations due to their association with increased mortality, longer hospital stays, and declines in functional status and cognition. Studies have shown that poor outcomes in the Intensive Care Unit, which are often linked with high scores in the Sequential Organ Failure Assessment (SOFA), are also correlated with frailty, as stated by Pan et al. [4]. In a study of patients undergoing coronary artery bypass graft (CABG) surgery, a relationship was found between prognosis based on SOFA scores and frailty status assessed by Fried’s scale. Patients were categorized into “favorable” and “unfavorable” prognosis groups according to their SOFA scores. As reported in the same study, 92.86% of patients in the unfavorable prognosis group (high SOFA scores) were classified as frail, compared to only 40.16% in the favorable group (low SOFA scores), with a statistically significant difference (P < 0.001). The unfavorable group demonstrated longer extubation times, extended hospital stays, and greater cognitive dysfunction postoperatively [4].

Frailty is present in a significant proportion of patients requiring surgical procedures. For example, a 2025 meta-analysis by Wu et al. synthesized data from 43 studies involving 14,441 elderly surgical patients [10]. The meta-analysis found that 34.0% (4,910/14,441) were frail preoperatively, and that 20% (2,783/14,441) developed postoperative delirium. This highlights the prevalence of frailty and its strong statistical association with postoperative delirium in older surgical populations; it also reinforces the need to apply frailty scales to predict surgical outcomes and delirium risk, as frailty itself is an independent risk factor. When a frailty phenotype is identified, a multidisciplinary approach is essential [10].

Despite the strong correlation between frailty and postoperative outcomes, frailty assessment scales remain largely underused. A study based on Italian surveys revealed that frailty scoring was performed in only 51.8% of care settings, and just 26.3% had a specific management pathway for elderly patients. Subjective assessment by anesthesiologists was the most used method, while more than half employed Enhanced Recovery After Surgery (ERAS) items, and fewer than 50% used delirium screening tools postoperatively. Most centers (73.7%) lacked formal policies for perioperative care of frail or older patients. Only 23.2% reported using the Edmonton Frail Scale, and 10.5% used the CGA. Approximately 30% indicated they assessed frailty only in very high-risk populations. Identifying frailty enables tailored intraoperative strategies regarding monitoring and anesthetic management. The study also highlights the importance of postoperative delirium screening, which remains infrequently performed [11].

Cognitive impairment and risk of postoperative delirium

Although cognitive impairment is highly prevalent among the elderly, baseline cognition is not routinely assessed. A 2023 meta-analysis by Au et al. [12] that analyzed 16 studies with a total of 62,179 patients found that preoperative cognitive impairment was associated with an eightfold increased risk of postoperative delirium in patients undergoing cardiac surgery, and a fourfold increased risk in those undergoing non-cardiac surgery. The proposed mechanism involves the disruption of an already impaired functional neural network, making delirium more likely. Therefore, recognizing and optimizing these risk factors preoperatively may help reduce delirium incidence. According to the ASA, preoperative cognitive screening occurs in fewer than 10% of surgical cases; nonetheless, routine neurocognitive testing would be beneficial [12].

Cognitive frailty is a subtype of frailty characterized by the coexistence of physical frailty and cognitive impairment without dementia; it reflects a cognitive state primarily driven by physical frailty [13]. Common cognitive screening tools include the Mini-Mental State Examination (MMSE) and the Montreal Cognitive Assessment (MoCA) [14]. Evidence indicates that cognitive frailty increases the risk of falls, disability, depression, and mortality, as described by Fang et al. [13]. Based on this, it has been hypothesized that cognitive frailty is also associated with a higher risk of postoperative complications and adverse outcomes. Studies support that frailty, cognitive frailty, and cognitive impairment are all linked to postoperative complications, with cognitive frailty conferring the highest risk [13]. 

Frequently reported complications in frail patients include delirium, loss of autonomy, infections, and cardiac and respiratory problems. These findings highlight the need for standardized diagnostic criteria to enable early identification and intervention [11,13]. A systematic review and meta-analysis by Jiang et al. [15], which included 15 studies with a total of 1,216 surgical patients, found that cognitive training significantly reduced the risk of postoperative cognitive dysfunction after noncardiac surgery (RR = 0.47; 95% CI: 0.29-0.76; P =  0.002). Improvements were also observed in executive function and memory [15].

Comprehensive Geriatric Assessment and multidimensional tools

Several tools are available for frailty assessment, but the CGA has been shown to be a superior predictive tool, as demonstrated by Theodorakis et al. [16]. It effectively identifies older patients at higher risk of postoperative delirium, infections, and prolonged hospital stays. The CGA evaluates frailty, sarcopenia, nutrition, cognition, mental health, and functionality, which are key aspects often affected in the elderly that contribute to perioperative complications. Commonly used tools may fail to accurately predict outcomes because they do not account for all these factors [16].

CGA encompasses medical comorbidities, polypharmacy, frailty, functional status, cognition, nutrition, risk of falls, and psychosocial factors. In patients with frailty, postoperative complications are five times more frequent, mortality is eight times higher, and delirium is three times more prevalent; these can be assessed using the scales previously mentioned. Sarcopenia is evaluated by grip strength or the chair stand test, followed by confirmatory assessments; it is also associated with increased complications, longer hospital stays, and greater need for rehabilitation. Malnutrition can be screened using the Mini Nutritional Assessment (MNA) and the Malnutrition Universal Screening Tool (MUST); it similarly increases complications and mortality. Cognitive dysfunction is assessed with the MMSE, MoCA, and Clock Drawing Test, and is linked to higher risks of complications, delirium, and mortality, as outlined by Theodorakis et al. [16]. Functional status impairment can be measured by the Barthel Index, Katz Index of Independence in Activities of Daily Living (ADL), or Lawton Index of Independence in Instrumental Activities of Daily Living (IADL). Impairments in these areas are associated with increased complications, delirium, and longer hospital stays [16].

The use of CGA can predict postoperative outcomes and identify candidates for prehabilitation and other interventions to improve prognosis. Frailty appears to be the most important predictor. However, because CGA is time-consuming, shorter screening tools (such as the G8 Geriatric Screening Tool) are often used to triage patients, reserving CGA for selected cases. As evidenced in different studies, routine frailty screening facilitates a better outcome prediction and tailored care planning for both the preoperative and postoperative period. The stronger recommendation would be to start the assessment with a frailty screening tool and move on to the CGA when frailty is determined in the previous steps [17]. This shorter version assesses factors including age, nutrition, weight, mobility, neuropsychological status, polypharmacy, and perceived health [16].

Recommended interventions in the perioperative period

Interventions based on assessment findings include nutritional management, exercise, physiotherapy, supplementation for deficiencies, and management of cognitive dysfunction and mental health disorders [16]. Nutritional interventions from seven to 14 days (the period could extend up to six weeks) help improve nutritional status, muscle mass, and postoperative recovery [18]. Narrative reviews emphasize including exercise, nutrition, and psychological support to increase physiological reserve prior to surgery [19].

Positive results have already been replicated, for instance, a study by Varley et al. [20] based on routine preoperative frailty assessment in which they followed the patients for a year after surgery demonstrated an 18% reduction in odds of one-year postoperative mortality (OR = 0.82; 95% CI: 0.72-0.92; P < 0.001) among over 50,000 elective surgical patients in a multihospital US healthcare system, following implementation of a Risk Analysis Index-based frailty screening initiative. The evaluation consisted of finding frail patients who needed to be referred for enhanced evaluation [20]. Another example corresponds to a prospective cohort in elderly cardiothoracic surgical patients; in this study, a multimodal prehabilitation resulted in improved walking distance and complication rates comparable to younger cohorts [21].

## Conclusions

Preoperative anesthesia assessment has followed a standardized approach for a long time; however, the shifting demographic demands a more tailored evaluation, particularly for elderly populations. Despite existing recommendations for optimizing perioperative care in older adults, implementation remains limited. While evidence on prehabilitation remains mixed, current findings are generally promising and support its use (particularly in frail patients) as part of a multidisciplinary strategy to improve postoperative outcomes.

In some settings, implementing a full CGA may be challenging; nonetheless, shorter screening tools can be used to identify high-risk patients, reserving the full CGA for selected cases. Early identification of frailty in the preoperative period enables cognitive and functional optimization or prehabilitation, which is likely to improve postoperative outcomes. Future research should focus on standardizing frailty screening protocols across surgical settings and conducting larger, well-powered trials to evaluate the effectiveness of cognitive prehabilitation in reducing postoperative complications.
